# The differences in microbial composition may be an important cause of pancreatic pseudocyst infection—an observational study based on 16SrRNA

**DOI:** 10.3389/fmicb.2025.1608297

**Published:** 2025-12-18

**Authors:** Yaoting Li, Yongzhan Zhao, Senlin Hou, Lichao Zhang

**Affiliations:** Department of Biliopancreatic Endoscopic Surgery, The Second Hospital of Hebei Medical University, Shijiazhuang, Hebei, China

**Keywords:** microorganism, pancreatic pseudocyst, infection, 16sRNA, EUS

## Abstract

**Background:**

There are no studies of pancreatic pseudocyst infections related to microorganisms. The purpose of this study was to analyze the microbiological differences between infective and non-infective pseudocysts.

**Methods:**

This was an observational cohort study. Thirty-seven patients with pancreatic pseudocyst who underwent EUS drainage at our center were included in the study. According to postoperative infection, the patients were divided into infected group and non-infected group. Capsular fluid was collected during endoscopic drainage and microbial sequencing was performed.

**Results:**

The clinical features of the two groups were similar (*p* > 0.05). There was no significant difference in *α* diversity between infected and non-infected groups (*p* > 0.05). There was significant difference in β diversity between infected and uninfected groups (Adonis, *R*^2^ = 0.039, *p* = 0.019). Random forest maps identified the top five species with the greatest abundance differences. At the genus level, the relative abundance of *Klebsiella, Streptococcus, Collinsella, Phascolarctobacterium*, and *Megamonas* in the sac fluid of infected group was significantly higher than that of non-infected group.

**Conclusion:**

The differences in the microbial composition of the cyst fluid in pancreatic pseudocyst may have an impact on postoperative infections. The relative abundance of *Klebsiella, Streptococcus, Collinsella, Phascolarctobacterium,* and *Megamonas* in infected group was significantly higher than that in noninfected group. Further research is still needed in the future to confirm this.

## Introduction

As a common complication of pancreatic diseases such as pancreatitis and pancreatic trauma, pancreatic pseudocysts are formed by the accumulation of pancreatic fluid, necrotic tissue, and inflammatory exudates around the pancreas, which become encapsulated by fibrous connective tissue to create cystic structures (Tan et al., 2021; Koo et al., 2024). Endoscopic transmural drainage is currently considered the first-line treatment for pancreatic pseudocysts (Li et al., 2020; Rasmussen et al., 2012). However, infection is a frequent complication following endoscopic drainage, adversely affecting patient prognosis and increasing medical costs (Guo et al., 2016). The etiology of infection after endoscopic transmural drainage of pancreatic pseudocysts remains poorly understood. Traditional views suggest that factors related to surgical intervention and the patient’s underlying conditions are key contributors to postoperative infection (Guo et al., 2016; Hentschel et al., 2022). However, growing evidence indicates that microorganisms may also play a significant role (Hentschel et al., 2022). The cyst fluid within pancreatic pseudocysts is not sterile, and the composition of its microbial community could influence the risk of postoperative infection. Previous studies have primarily focused on macroscopic clinical factors associated with infection, while the microscopic microbiological environment within the cyst fluid has remained largely unexplored. Therefore, this study aims to characterize the microbial composition of pseudocyst fluid using 16S rRNA gene sequencing and to investigate its potential association with post-procedural infection, with the goal of identifying microbial factors that may contribute to infection following endoscopic transmural drainage of pancreatic pseudocysts.

## Materials and methods

### Study design

This study was designed as an observational investigation to explore the role of microorganisms in infections following endoscopic transmural drainage of pancreatic pseudocysts. The study protocol was approved by the Institutional Review Committee of our hospital. Patients with pancreatic pseudocysts were consecutively recruited from the Department of Biliary and Pancreatic Endoscopic Surgery. Written informed consent was obtained from all participants prior to enrollment. All study data were made accessible to the patients. We confirm that all procedures were performed in accordance with relevant guidelines and regulations.

### Selection criteria

#### Inclusion criteria

1. Patients diagnosed by enhanced CT or MRI with pancreatic pseudocyst formation with a mature cyst wall.
2. The patient had indications of endoscopic drainage, including pain, bloating, nausea, and jaundice, but no infection.
3. Fluid accumulation lasting more than 6 weeks, conservative treatment is not effective.

#### Exclusion criteria

1. Patients with spontaneous infection or spontaneous bleeding in the effusion.
2. Patients with contraindications such as portal hypertension and gastrointestinal bleeding.
3. Refuse to participate in the study.
4. Patients with related diseases such as coagulation dysfunction or diabetes that may affect autoimmunity.

### Study groups

According to postoperative infection, patients were divided into infected group and non-infected group. The capsule fluid was collected during endoscopic transmural drainage and microbial sequencing was performed.

### Procedure and sample collection

All patients underwent routine blood routine and blood biochemistry examination after admission. Blood routine and procalcitonin tests were performed on the 1st and 3rd day after surgery to determine the inflammation and infection. The infection group is defined as an increase in white blood cells or procalcitonin accompanied by a fever greater than 38.5 °C. The drainage of pancreatic pseudocyst was evaluated by CT 1 week after surgery. Take your temperature at least twice a day to assess your fever. All endoscopic procedures are performed by experienced endoscopists. Each surgical procedure adheres to strict aseptic techniques, and all experiments and surgical materials are subjected to strict sterilization procedures. Endoscopic ultrasound (OLYMPUS, JAPAN) was used to accurately scan pancreatic pseudocysts to ensure the exclusion of bleeding and solid nodules. The ECHO-19 puncture needle (COOK) is then used to puncture at a suitable location (stomach or duodenum) that avoids blood vessels. Once the needle is confirmed to be inside the cyst, 5–10 mL of cyst fluid is extracted using a sterile negative pressure syringe. The collected liquid is then placed in a sterile test tube and immediately stored in a refrigerator at −80 °C for subsequent microbiological analysis.

#### Primary outcome

Primary outcome measures focused on differences in the abundance and species of microorganisms in the cyst fluid between the postoperative infected and non-infected groups.

### Metabarcoding of cyst fluid

Soil DNA kit (Omega) was used to extract genomic DNA from peripancreatic effusion samples. The DNA was measured by 0.8% agarose gel electrophoresis and quantified by ultraviolet spectrophotometer. The DNA is stored in a −20 °C refrigerator for subsequent analysis after passing the test. In order to ensure the sequencing quality, the optimal insertion segment for sequencing was 200–450 bp according to the Miseq sequence read length. In this study, V3 ~ V4 regions of 16S rRNA gene were selected for sequencing. The PCR primers were 338F (5′-ACTCCTACGGGAGGCAGCA-3′) and 806R (5′-GGACTACHVGGGTWTCTAAT-3′). Conventional PCR thermal cycle procedures: initial denaturation at 98 °C for 30 s, denaturation at 98 °C for 15 s, annealing at 50 °C for 30 s, extension at 72 °C for 30 s. The above three processes were carried out for 25 cycles, finally extended for 5 min at 72 °C, and finally stored conventionally at 4–10 °C. Amplification results were performed on 2% agarose gel electrophoresis. After the detection was qualified, the target fragment was removed, and the target fragment was recovered with the oxygen gel recovery kit. The PCR products were quantified using the Quant-iTPico Green dsDNA Assay Kit on a Microplate reader (BioTek, FLx800) and then mixed according to the amount of data required for each sample. Illumina truseq was used to construct sequencing library of the PCR products obtained. Qualified sequencing library (index sequence cannot be repeated) was diluted according to gradient, then mixed according to sequencing amount, denatured into single strand with NaOH, and sequenced on the machine. The barcode V3–V4 amplicon was sequenced by Illumina Miseq. 200–450 bp was selected as the best sequencing length. Preliminary screening was conducted according to overlapping bases, and the pairing of the peer sequences was performed using FLASH software: the overlapping base lengths of Read 1 and Read 2 sequences were required to be ≥10 bp, and base mismatch was not allowed. Then the valid sequence is obtained according to the Index information corresponding to each sample. Finally, QIIME software (Quantitative Insights Into Microbial Ecology, v1.8.0) was used to identify error sequences and remove them. The amplification and sequencing of 16S rRNA gene was completed by Personal Biotechnology Co., LTD. (Shanghai).

### Statistical analysis

The main outcome of our study was to observe the difference of microbiota in the two groups of cyst fluid. Statistical analyses were performed using IBM SPSS 27.0 and R 3.4.4 with a test level of *α* = 0.05. Alpha diversity was analyzed using QIIME2 (2019.4), R language, ggplot2 package, and Kruskal–Wallis rank sum test and Dunn’s test as *post hoc* tests to verify the significance of differences. Shannon index, Simpson index, Chao1 index and observed species index were used to analyze the *α* diversity between the two groups. The beta diversity was analyzed using scikit-bio package, R language, vegan package implementation. The Adonis method was used to analyze the differences in beta diversity between groups. The explainability (*R*^2^) and significance (*p*) of the grouping scheme to the variance of the distance matrix were calculated through the vegan package of *R*, and the number of permutation tests was set to 999.

## Result

### Patient characteristics

The study included a total of 37 patients with pancreatic pseudocysts who were treated at our department between June 2022 and June 2023. Based on infection status, the cohort was divided into an infected group (*n* = 18) and a non-infected group (*n* = 19). The mean age was 46.4 years in the infected group and 49.7 years in the non-infected group. Gender distribution consisted of 13 males and 5 females in the infected group, and 12 males and 7 females in the non-infected group.

### Comparison of general clinical data between infected group and non-infected group

Table 1 presents general clinical data for the infected and non-infected groups. There were no significant differences in gender, age, cyst location, and cyst diameter between the two groups (*p* > 0.05).

**Table 1:** Demographic and clinical characteristics of infection vs. non-infection groups.

Variable	Non-infection group (*n* = 19)	Infection group (*n* = 18)	*p*-value
Gender (male)	12 (63.2%)	13 (72.0%)	0.751
Age (years)	46.4 ± 13.9 (45)	49.7 ± 19.6 (48.5)	0.601
Cyst location	–	–	0.614
Body/tail	18 (94.7%)	15 (83.3%)	–
Pancreatic head	1 (5.3%)	3 (16.7%)	–
Cyst diameter (cm)	11.2 ± 2.3 (11)	9.3 ± 2.8 (9.6)	0.285

### OTU level analysis

**Figure 1:**
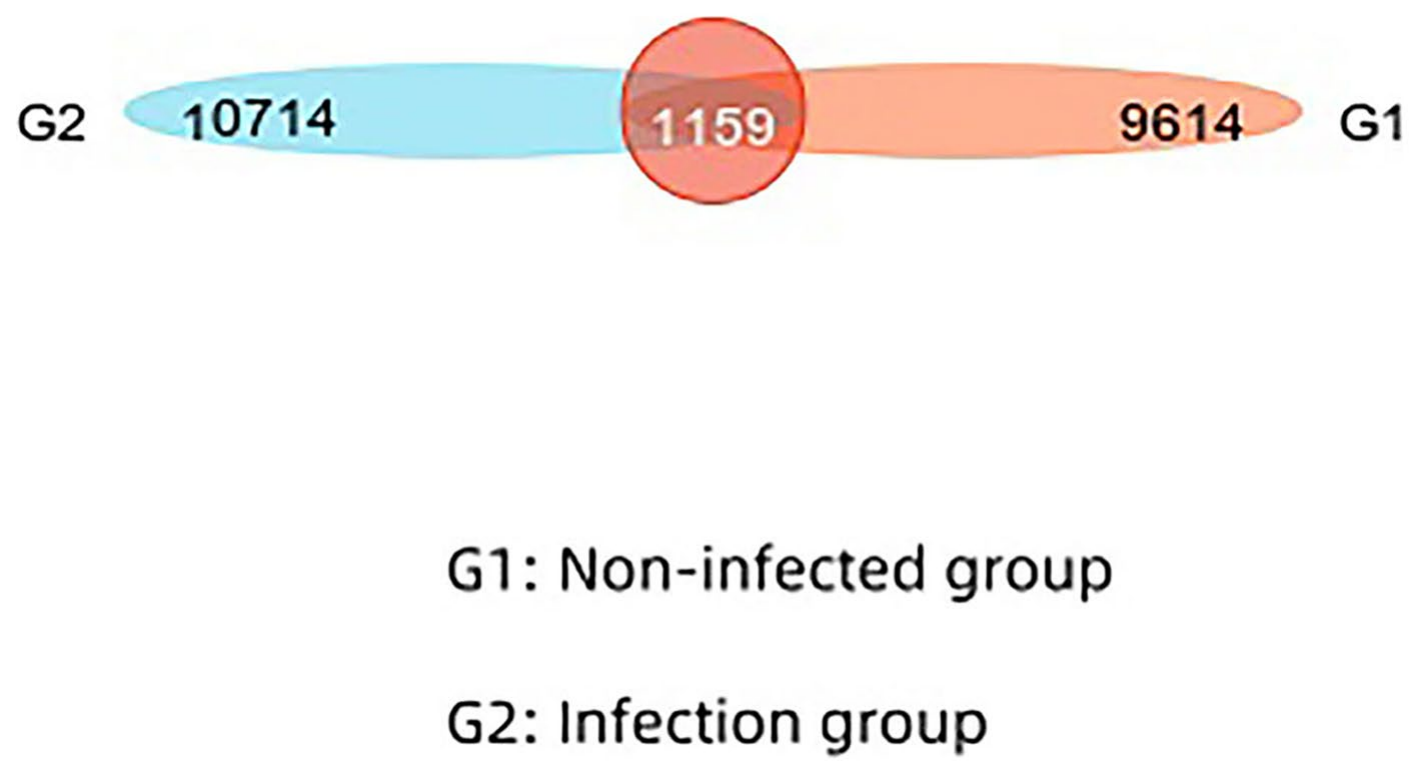
Similarities and overlaps in the number and composition of OTUs in infected and non-infected groups are visualized in the Venn diagram.

In this study, we performed Operational Taxonomic Unit (OTU) clustering of non-repetitive sequences (excluding single sequences) based on 97% similarity, and removed chimeras in the clustering process to obtain representative OTU sequences. The total number of OTU in the two groups was 20,328. The number of overlapping OTUs between the two groups was 1,159. The similarity and overlap of the number and composition of OTUs for each group can be visualized in the Venn diagram (Figure 1).

### Species composition analysis

**Figure 2:**
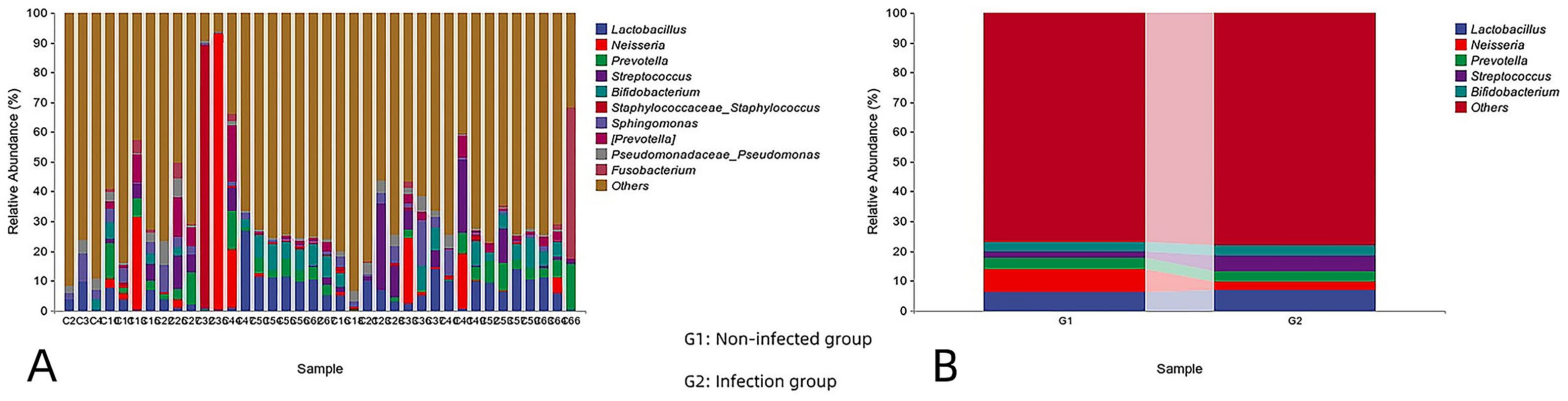
**(A)** Relative abundance of the top 10 bacterial taxa genera in a single sample. **(B)** The bar chart shows the average relative abundance of the top 5 genera of each bacterial group.

**Figure 3:**
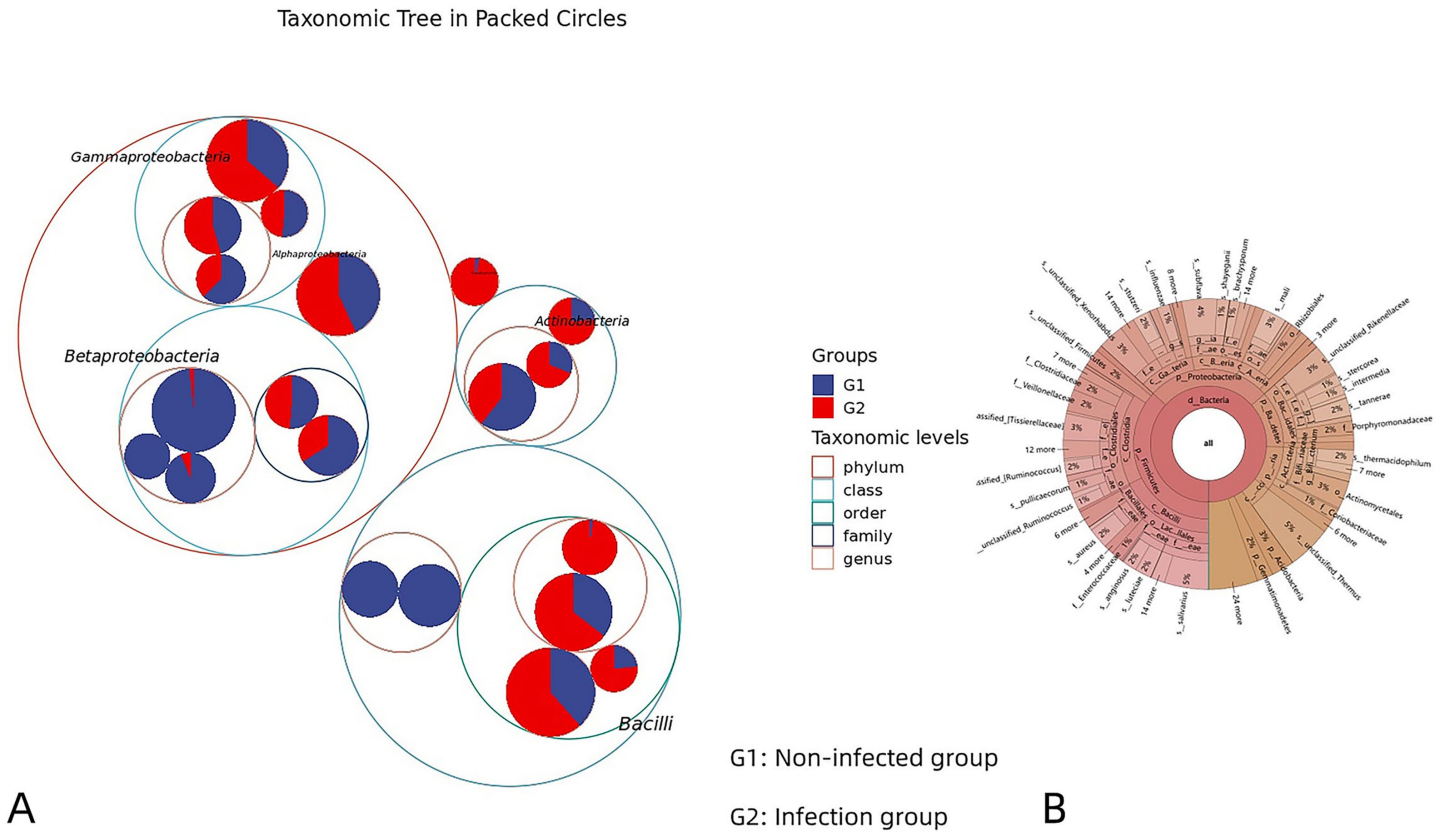
**(A)** Taxonomic differences based on 16S rRNA sequences extracted from metagenomes. **(B)** The krona circle diagram represents seven taxonomic levels from inside to outside: domain, phylum, class, order, family, genus, and species, and the size of the fan reflects the relative abundance of different taxa.

The relative abundance of each taxonomic unit at every classification level was calculated based on the proportion of sequences assigned to each OTU. Analysis of individual samples revealed that the most prevalent intestinal bacterial genera in both infected and non-infected groups included *Lactobacillus*, *Neisseria*, *Prevotella*, *Streptococcus*, *Bifidobacterium*, Staphylococcaceae, *Staphylococcus*, *Sphingomonas*, Pseudomonadaceae, *Pseudomonas*, and *Fusobacterium*. Figure 2A illustrates the relative abundance of the top 10 genera within a representative sample. Figure 2B compares the average relative abundance of the five most abundant genera in each group. In the non-infected group, the dominant genera were *Lactobacillus* (6.29%), *Neisseria* (7.61%), *Prevotella* (4.01%), *Streptococcus* (1.98%), and *Bifidobacterium* (3.11%). Notably, *Neisseria* (7.61%) exhibited the highest abundance in the cyst fluid of non-infected patients (G1). In contrast, the infected group (G2) showed high relative abundances of *Lactobacillus* (6.91%), *Neisseria* (2.73%), *Prevotella* (3.59%), *Streptococcus* (5.25%), and *Bifidobacterium* (3.46%), with *Lactobacillus* (6.91%) being the most abundant genus. The taxonomic hierarchy tree (Figure 3A) and the Krona chart of species composition (Figure 3B) provide a more intuitive visualization of the microbial community structures in both the infected and non-infected groups.

### Alpha diversity analysis

**Figure 4:**
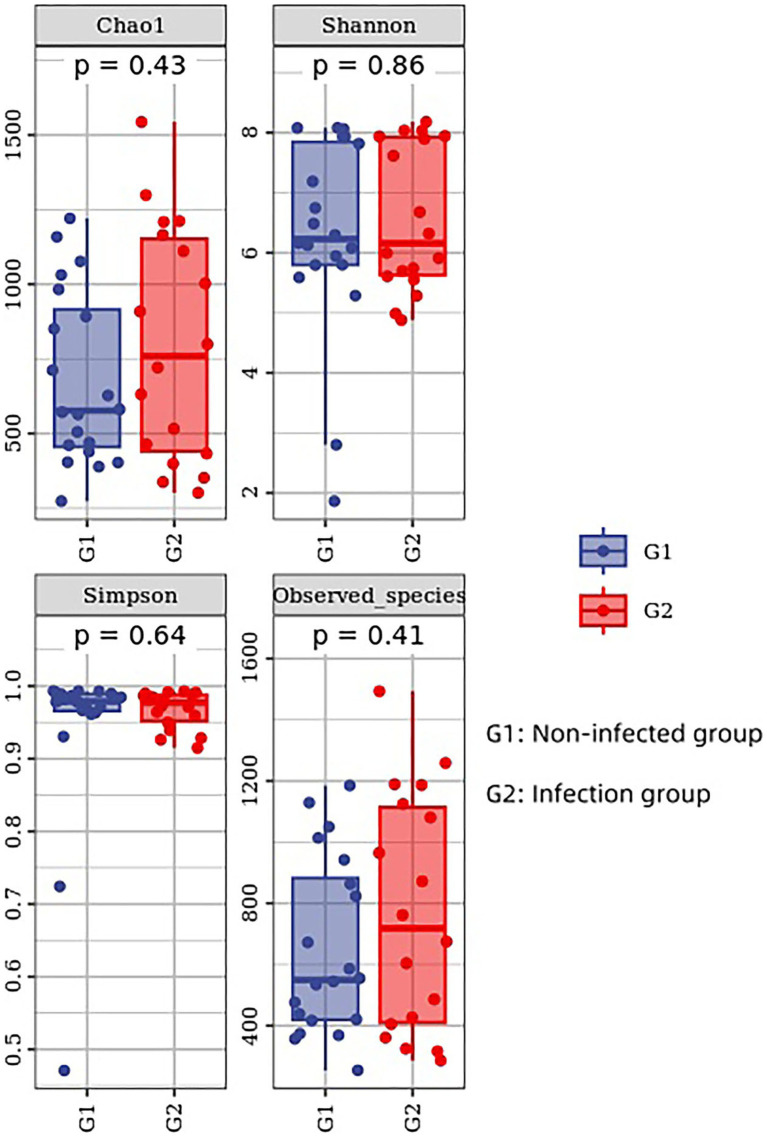
The diversity index of Chao 1, Simpson, Shannon and observed species index was used to reflect the *α* diversity.

The diversity index of Chao 1, Simpson, Shannon, and observed species index was used to reflect the *α* diversity. The results of Shannon, Simpson, Chao 1, and observed species index showed that the levels of microbial diversity were similar between the infected group and the non-infected group, with no statistically significant difference (*p* > 0.05) (Figure 4). This suggests that there was no significant increase in microbial richness and diversity in the infected group compared to the non-infected group.

### Beta diversity analysis

**Figure 5:**
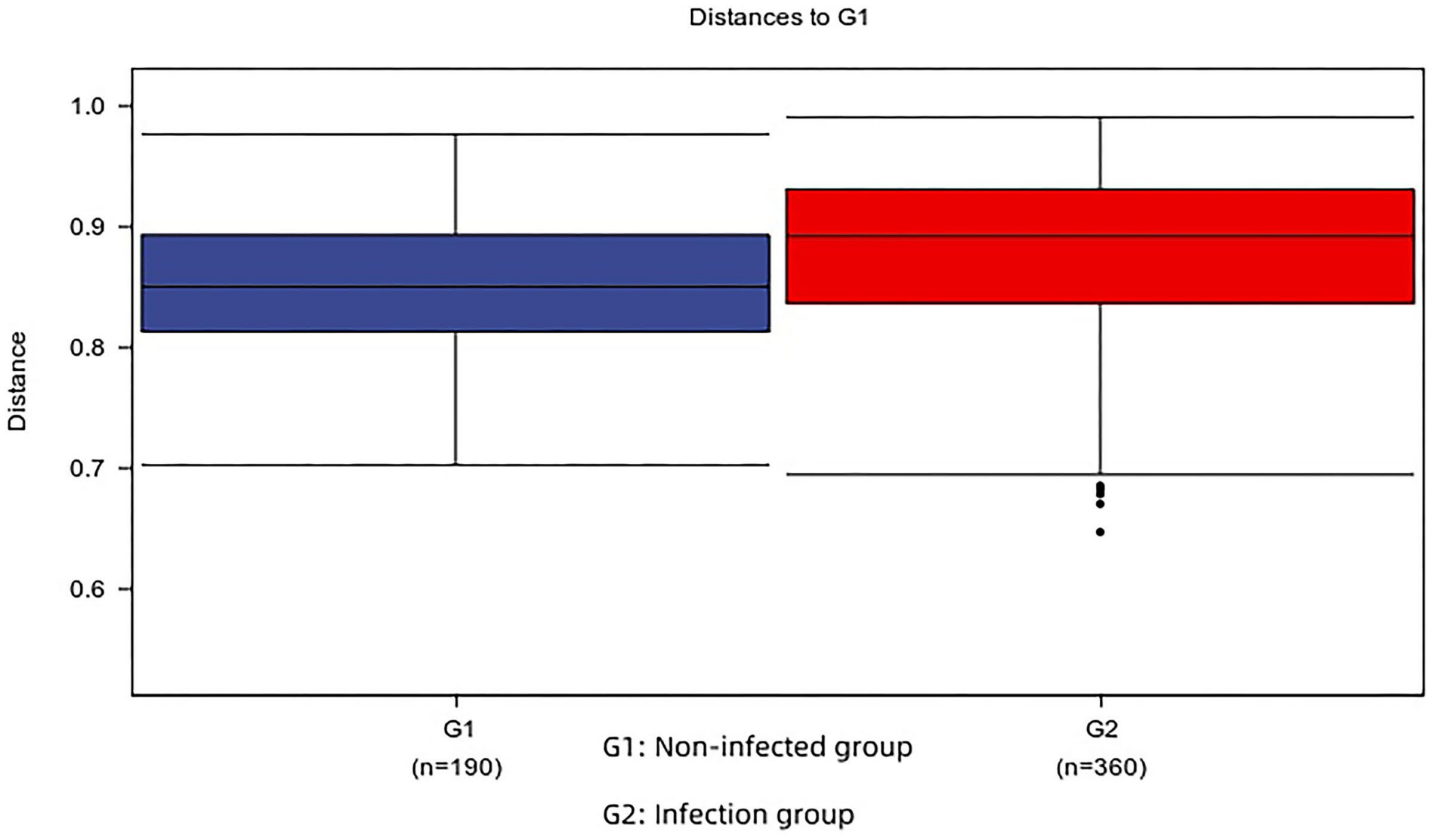
The boxplot reflects the difference in *β* diversity between the two groups. The upper and lower end lines of the box diagram are Interquartile range (IQR). The median line is the median; the upper and lower edges are the maximum and minimum values; the points outside the upper and lower edges represent outliers.

**Figure 6:**
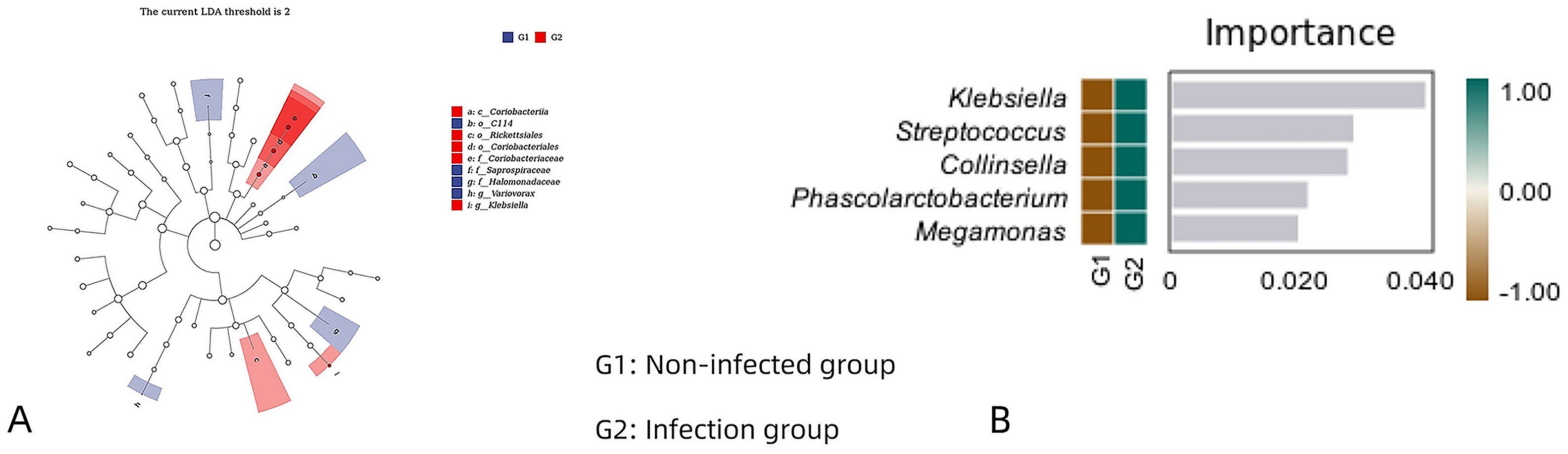
**(A)** Taxonomic branching diagram shows the taxonomic hierarchy of the major taxa in a sample community from phylum to genus (inner circle to outer circle). The node size corresponds to the average relative abundance of the taxon. Hollow nodes represent taxa that do not differ significantly between groups, while nodes of other colors (such as green and red) indicate that these taxa exhibit significant differences between groups and are more abundant in the sample represented by that color. Letters identify the names of taxa that differ significantly between groups. **(B)** The horizontal coordinate of the bar chart marks the importance of species to the taxonomic model, and the vertical coordinate is the taxonomic unit name at the genus level. The importance of species to the model decreases from top to bottom.

The structural differences between the infected and non-infected groups were analyzed by *β*-diversity. Unweighted UnifRAC-based PCoA revealed differences in microbial composition between infected and non-infected groups. The results were further confirmed by Adonis, which showed a significant difference in microbiome composition between the infected and non-infected groups (Adonis, *R*^2^ = 0.039, *p* = 0.019) (Figure 5). Although the *p*-value was statistically significant (*p* = 0.019), the *R*^2^ value was low (0.039), indicating that infection status explains only 3.9% of the variance. LEfSe tests were used to further evaluate different taxa of sac fluid from infected and non-infected patients (Figure 6A). Random forest maps identified the top five species with large abundance differences (Figure 6B). Interestingly, at the genus level, the relative abundance of *Klebsiella, Streptococcus, Collinsella, Phascolarctobacterium,* and *Megamonas* in the infected group is significantly higher than that in the non-infected group. LEFSe and random forest analysis did not apply corrections for multiple comparisons, which might increase the risk of false positive results.

## Discussion

Postoperative infection is a serious complication of endoscopic transmural drainage for pancreatic pseudocysts, adversely affecting patient prognosis. The etiology is multifactorial, involving cyst size, drainage adequacy (Guo et al., 2016), and the patient’s immune status (Hentschel et al., 2022). Theoretically, microorganisms within a pseudocyst are confined by the cyst wall and seldom provoke an immune response. However, drainage exposes cyst contents to the immune system, potentially triggering clinical infection marked by fever and leukocytosis. This study therefore aimed to compare cyst fluid microbiota between infected and non-infected pseudocysts. Prior microbiological analyses have largely relied on fluid culture (Hentschel et al., 2022; Schepis et al., 2021; Parida et al., 2014; Isaji et al., 2003), which has inherent limitations in capturing full microbial diversity. In contrast, we employed 16S rRNA sequencing to comprehensively profile the microbiota in pancreatic pseudocyst (PPC) fluid after endoscopic drainage, comparing postoperative infected and non-infected cases. Our findings provide novel insights into the microbiology of post-drainage infections and have implications for clinical management.

We enrolled 37 patients—18 infected and 19 non-infected—with no significant differences in baseline characteristics (*p* > 0.05). Microbial composition analysis showed that both groups were dominated by *Lactobacillus*, *Neisseria*, *Prevotella*, *Streptococcus*, and *Bifidobacteriu-m*, indicating non-sterility of cyst fluid even in asymptomatic cases, likely due to bacterial translocation during pancreatitis. Alpha diversity was similar between groups, suggesting infection was not driven by increased microbial richness. However, beta diversity revealed a significant compositional difference (Adonis, *R*^2^ = 0.039, *p* = 0.019). LEfSe identified differentially abundant taxa, though the low *R*^2^ value and lack of multiple-testing correction limit interpretability. Random forest analysis highlighted five genera with the greatest abundance differences: *Klebsiella, Streptococcus, Collinsella, Phascolarctobacterium, and Megamonas* were all enriched in infected cases. *Klebsiella*, a gut commensal and opportunistic pathogen (Blenn and Beyaert, 2021), has been isolated in chronic pancreatitis (Parida et al., 2014). It can activate pancreatic plasmacytoid dendritic cells and M2 macrophages via microbial-associated molecular patterns, inducing IFN-α and IL-33, potentially linked to autoimmune pancreatitis (Kamata et al., 2019). Its capsule aids immune evasion and cystic proliferation. *Streptococcus*, enriched in the gut in mild pancreatitis (Yu et al., 2021), may act opportunistically in pseudocysts. *Collinsella* adheres to mucosa via adhesins, releases endotoxins, and may promote systemic inflammation; it also modulates the endocannabinoid system and correlates with lymphocyte counts (Vijay et al., 2021). Further mechanistic studies are needed to establish causality.

Our results indicate that *Klebsiella*, *Streptococcus*, and *Collinsella* are significantly enriched in infected cyst fluid. These genera are often implicated in pancreatitis-related infections, with *Klebsiella* particularly correlated with clinical outcomes, suggesting their critical role in post-drainage infection. The utility of prophylactic antibiotics in PPC drainage remains debated. A recent randomized trial suggested they may be unnecessary in sterile pseudocysts with adequate drainage (Jagielski et al., 2022). However, the enrichment of opportunistic pathogens in infected cases in our study supports further investigation into targeted antibiotic prophylaxis.

To our knowledge, this is the first study to characterize the PPC fluid microbiome and identify specific microbiota associated with postoperative infection. Limitations include a small sample size and single-center design, which may affect generalizability. Demographic and regional treatment variations could also influence microbiome-infection relationships. Larger, multi-center studies are needed to validate these findings.

Although 16S rRNA sequencing effectively profiles bacteria, it does not detect viruses, fungi, or other microbes. The PPC fluid microbiome is a complex ecosystem involving multi-kingdom interactions. Future studies using metagenomic, metatranscriptomic, and metabolomic approaches will be essential to fully understand microbial networks, host-microbe crosstalk, and metabolic pathways, ultimately informing effective strategies for preventing postoperative infection.

## Conclusion

The differences in the microbial composition of the cyst fluid in pancreatic pseudocyst may have an impact on postoperative infections. The relative abundance of *Klebsiella, Streptococcus, Collinsella, Phascolarctobacterium, and Megamonas* in infected group was significantly higher than that in non-infected group. Further research is still needed in the future to confirm this.

## Data Availability

The raw data supporting the conclusions of this article will be made available by the authors, without undue reservation.
